# Supplementary material for: Rotational Barriers in N-Benzhydrylformamides: An NMR and DFT Study
Source: Molecules. 2023 Jan 5;28(2):535. doi: 10.3390/molecules28020535 (PMC9863877; doi:10.3390/molecules28020535)
Supplement: Supplementary file 1 [file molecules-28-00535-s001.zip › Supplementary_Materials/Supplementary_Table_S1.pdf]

Supplementary Materials

Rotational Barriers in N-Benzhydrylformamides: An NMR and DFT Study

Madina Zh. Sadvakassova[1]*, Andrei I. Khlebnikov[2], Abdigali A. Bakibaev[3], Oleg A. Kotelnikov[3], Rakhmetulla Sh. Erkassov[1], Madeniyet A. Yelubay[4] and Manar A. Issabayeva[4]

[1] Department of Chemistry, L.N. Gumilyov Eurasian National University, 010008 Astana, Kazakhstan

[2] Kizhner Research Center, Tomsk Polytechnic University, 634050 Tomsk, Russia

[3] Faculty of Chemistry, National Research Tomsk State University, 634050 Tomsk, Russia

[4] Department of Chemistry and Chemical Technology, Toraighyrov University, 140008 Pavlodar, Kazakhstan

* Correspondence: madinas-t@mail.ru; Tel.: +7-777-9729555

Table S1. Relative Gibbs free energies of the conformations obtained after DFT optimization (M062X/6-311+G*/IEFPCM(Dimethylsulfoxide)) of the structures initially generated by VConf program of VeraChem package[a].

Compound	Conformation	$\Delta G°_{298}$, kcal/mol	Compound	Conformation	$\Delta G°_{298}$, kcal/mol
BHFA	1	1.318	BHFA-oCl	1	1.677
	2	1.390		2	2.307
	3	1.163		3	1.694
	4	1.071		4	2.482
	5	1.384		5	3.028
	6	1.418		6	2.214
	7	1.466		7[b]	0.000
	8	1.260		8[b]	1.348
	9	1.230		9	1.548
	10[b]	0.000		10	2.302
BHFA-NMe	1[b]	0.000	BHFA-oBr	1	1.755
	2[b]	0.049		2	2.423
	3[b]	0.056		3	1.606
	4[b]	0.089		4	2.717
	5	0.091		5	3.178
	6	0.143		6	2.507
	7	0.152		7[b]	0.000
	8	0.153		8	1.759
	9[b]	0.200		9	1.923
	10[b]	0.215		10	2.480
BHFA-oF	1	1.835	BHFA-oI	1	1.726
	2	1.699		2	3.091
	3	1.595		3	1.701
	4	1.700		4	2.361
	5[b]	0.000		5	3.032
	6	1.487		6[b]	0.000
	7[b]	0.771		7[b]	1.606
	8	1.604		8	2.319
	9	1.733		9[b]	0.071
	10	1.613		10	1.740

[a] The Gaussian output files were archived and attached to Supplementary Materials.
[b] Conformation with *syn*-orientation of the formyl and benzhydryl moieties.
